# Lineup position affects guessing-based selection but not culprit-presence detection in simultaneous and sequential lineups

**DOI:** 10.1038/s41598-024-78936-9

**Published:** 2024-11-12

**Authors:** Carolin Mayer, Raoul Bell, Nicola Marie Menne, Axel Buchner

**Affiliations:** Department of Experimental Psychology, Faculty of Mathematics and Natural Sciences, Heinrich Heine University Düsseldorf, Universitätsstraße 1, 40225 Düsseldorf, Germany

**Keywords:** Position effects, Simultaneous lineups, Sequential lineups, Two-high threshold eyewitness identification model, Psychology, Human behaviour

## Abstract

The two-high threshold eyewitness identification model was applied to investigate the effects of lineup position on the latent cognitive processes underlying eyewitness responses in lineups. In two experiments with large sample sizes and random assignment of culprits and innocent suspects to all possible lineup positions, we examined how detection-based and non-detection-based processes vary across the positions of six-person photo lineups. Experiment 1 (*N* = 2586) served to investigate position effects in simultaneous lineups in which all photos were presented in a single row. Experiment 2 (*N* = 2581) was focused on sequential lineups. In both experiments, lineup position had no effect on the detection of the presence of the culprit. Guessing-based selection, in contrast, differed as a function of lineup position. Specifically, a lineup member placed in the first position in a lineup was significantly more likely to be selected based on guessing than lineup members placed in other positions. These results justify the practice of avoiding to place the suspect in the first position in a lineup, as this placement increases the suspect’s chance of being selected based on guessing.

## Introduction

Prompted by concerns that false eyewitness identifications can lead to wrongful convictions^1^, researchers have investigated influences on eyewitness performance for decades^2–11^. In some cases, their insights have made their way into practice^12^, and some of their findings are now included in the recommended procedures in different jurisdictions^2,13^. In other instances, it is unclear whether existing practice in applied contexts is corroborated by scientific research. Specifically, the placement of suspects in lineups sometimes conflicts with formal recommendations. On the one hand, the Technical Working Group for Eyewitness Evidence has recommended randomly selecting the suspect’s position for each lineup^14^. On the other hand, practitioners often avoid placing the suspect in certain positions when they compile lineups. In a survey by Wogalter et al.^15^, over 80 % of police officers reported usually placing the suspect in the middle of a lineup. Additionally, an analysis of case files showed that the suspect had been placed in Positions 2 to 5 in about 85 % of the cases, leaving only 15 % of the cases in which the suspect had been placed in Positions 1 or 6 combined^16^. In line with police practice, lineup researchers sometimes avoid placing the suspect in the first or last position in lineups as well^17^. Referring to sequential lineups in particular, researchers have expressed concerns with placing the suspect in the first position. For instance, Malpass et al.^18^ have recommended that, in addition to randomizing positions, “the suspect should not be presented first in the sequence” (p. 4) to reduce the rate of false identifications. There are also reports that placing the suspect in the first position may give the defense opportunity to argue against the validity of the lineup procedure^2,19,20^. To illustrate, Wells et al.^2^ write: “Valid concerns were raised (…) about a situation in which the suspect was placed in Position 1 (positioning is supposed to be random) and the eyewitness selects the suspect. The defense would argue that this was the equivalent of a showup (an identification procedure with only the suspect and no fillers)” (p. 595). In consequence, presenting the suspect in first position has been avoided in field studies^19,20^. Considering these widespread concerns about placing the suspect in the first position in a lineup, the question arises as to whether these concerns are in fact scientifically valid.

Researchers have employed various performance measures to investigate lineup-position effects, such as response rates^21–23^, probative value^24,25 ^or ROC-based analysis of pAUCs^26–29^, typically with the aim of determining in which position the guilt or innocence of a suspect can be best ascertained by a specific procedure such as the sequential lineup. A thorough review of the literature on possible lineup-position effects on eyewitness performance has shown that the empirical results are mixed and inconclusive^28^. A common limitation of many previous studies lies in the selective and non-random assignment of the lineup members to positions. For example, the suspect may have been placed in Positions 2 and 5, with no regard to Position 1. Furthermore, the interpretation of existing results is often complicated by small sample sizes, reducing the chances of finding a position effect if it exists.

In the present study, we examined lineup-position effects on the latent cognitive processes underlying eyewitness responses using large sample sizes and random assignment of culprits and innocent suspects to all lineup positions. To this end, the two-high threshold (2-HT) eyewitness identification model^30,31 ^was applied to the analysis of position effects. The model belongs to the general class of multinomial processing tree models which serve to estimate the probabilities of latent cognitive processes from behavioral data^32–34^. In memory research, these models have become popular due to their capacity to separate various types of memory processes from each other and from guessing processes^35–41^. To separately measure the cognitive processes underlying eyewitness responses, the 2-HT eyewitness identification model incorporates all observable response categories that can emerge in lineups: suspect identifications, filler identifications and lineup rejections in both culprit-present and culprit-absent lineups. Based on the frequencies of responses in these response categories, parameters are estimated that represent the detection of the presence of the culprit, the detection of the absence of the culprit, biased selection of the suspect and guessing-based selection of a lineup member. A crucial advantage of this measurement model is that its parameters have been rigorously validated, both in studies explicitly designed to test the model’s validity^30 ^and in reanalyses of data from other laboratories^31^. Additionally, the model has already proven to be highly useful for investigating novel research questions about the latent processes underlying eyewitness responses^5,42–45^.

**Figure 1:**
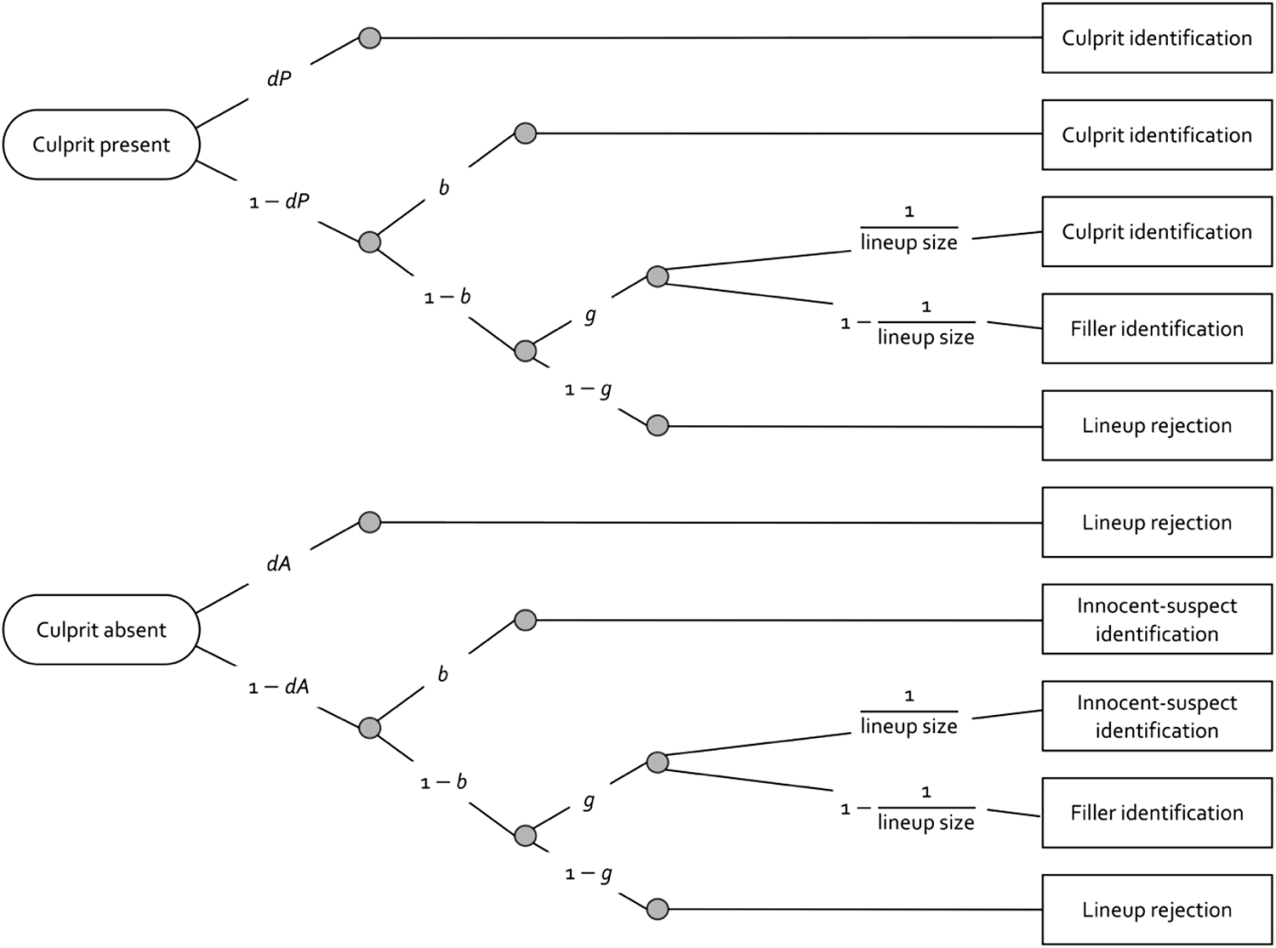
An illustration of the 2-HT eyewitness identification model in the form of processing trees. The ovals on the left represent the different types of lineups presented: culprit-present lineups and culprit-absent lineups. The rectangles on the right show the observable response categories. The letters attached to the branches connecting the ovals and rectangles represent the cognitive processes underlying eyewitness responses (*dP*: probability of detecting the presence of the culprit; *b*: probability of biased selection of the suspect; *g*: probability of guessing-based selection of a lineup member; *dA*: probability of detecting the absence of the culprit). Guessing-based selection results in the selection of the suspect with the sampling probability that is given by the reciprocal of the lineup size.

An illustration of the 2-HT eyewitness identification model is displayed in Fig. 1. This model is a joint multinomial model^34 ^comprising two trees. The upper tree depicts the processes that may occur when the culprit is present in the lineup. The lower tree depicts the processes that may occur when the culprit is absent. Since the model is a joint multinomial model, data from both culprit-present and culprit-absent lineups are used to estimate all four parameters of the model^46^.

In a culprit-present lineup, the presence of the culprit is detected with probability *dP*, resulting in a correct identification. If the presence of the culprit is not detected, which occurs with the complementary probability 1$\:-$* dP*, the eyewitness’s responses are determined by non-detection-based processes. With the conditional probability *b*, the eyewitness may select the suspect because the suspect stands out from the fillers; this may be the case in unfair lineups^30,31^. In case of no biased suspect selection, which occurs with the complementary probability 1$\:-$* b*, the eyewitness may select a lineup member based on guessing. Guessing-based selection occurs with the conditional probability *g*. In this case, the conditional probabilities of suspect and filler identifications depend upon the lineup size. With probability 1* lineup size* ($\:\text{1}\stackrel{\text{-}}{\text{6}}$ 1 ÷ 6 in six-person lineups), the lineup member selected based on guessing is the suspect. With probability 1$\:-$ (1$\:\div$* lineup size*), the lineup member selected based on guessing is a filler. In case of no guessing-based selection, which occurs with the complementary probability 1$\:-$* g*, the eyewitness may reject the lineup.

If the culprit is not present in the lineup, their absence is detected with probability *dA*, leading to a correct rejection of the lineup. If the absence of the culprit is not detected, which occurs with the complementary probability 1 $\:-$* dA*, the same non-detection-based processes take place as in culprit-present lineups. These non-detection-based processes are the same in both culprit-present and culprit-absent lineups because, when neither the presence nor the absence of the culprit is detected, culprit-present and culprit-absent lineups appear the same to the eyewitness.

In previous applications of the 2-HT eyewitness identification model^5,42–45^, the analyses were focused on data aggregated over lineup positions. Here, we applied the 2-HT eyewitness identification model to the examination of how lineup position affects the cognitive processes underlying eyewitness responses. Lineup-position effects were examined in simultaneous lineups in Experiment 1 and in sequential lineups in Experiment 2. Accordingly, the results of previous research regarding position effects in simultaneous and sequential lineups are discussed in the introductions to Experiments 1 and 2, respectively.

## Experiment 1

Experiment 1 was designed to investigate the effects of lineup position on the detection-based and non-detection-based processes underlying eyewitness responses in simultaneous lineups. In simultaneous lineups, all lineup members—the suspect and the fillers—are presented at the same time^47^. The lineups used here consisted of six photos presented in a single row, with Position 1 referring to the leftmost position and Position 6 referring to the rightmost position.

The first aim of Experiment 1 was to test whether lineup position affects the detection of the presence of the culprit. For most previous studies it was reported that lineup position had no effect on the rate of culprit identifications or the ability to discriminate between culprits and innocent suspects in simultaneous lineups^6,28,48^. However, O’Connell and Synnott^22^ reported better discriminability in middle positions which could come about if participants focused their attention more strongly on middle positions than on edge positions. Based on these findings, it is interesting to test whether culprit-presence detection is increased in middle positions of a lineup even though the results of most studies suggest that there should be no effect of lineup position on the detection of culprit presence in simultaneous lineups.

Another aim of Experiment 1 was to test whether lineup position affects guessing-based selection. For several previous studies it was reported that lineup position did not affect overall identification rates, rejection rates or the response criterion in simultaneous lineups^20,28,48^. However, there are also studies suggesting that people may be more likely to select lineup members based on guessing in earlier positions^21,49,50 ^or in middle positions^21^. In summary, there is thus some evidence in support of the concerns about placing the suspect in Position 1 in a lineup^2,19,20^. The purpose of the present experiments was to assess, using sensitive statistical tests based on large sample sizes, whether the reason for these concerns can be confirmed or not.

### Method

#### Participants

Participants were recruited and compensated through the research panel of Bilendi GmbH (https://www.bilendi.de). Data for Experiments 1 and 2 were collected simultaneously in one wave for logistic and monetary reasons. Of the data files of the participants who filled out the socio-demographic questionnaire at the beginning of Experiment 1, a total of 443 were excluded because the participants either did not complete the experiment or revoked the consent to the use of their data, 149 were excluded because the participants saw the staged-crime video more than once and 51 were excluded because the participants failed the attention check (see Procedure section). The final sample included 2586 participants (1193 female, 1389 male, 4 diverse) with a mean age of 54 years (*SD* = 16). The sample was characterized by a diversified level of education: Secondary education had been completed by around 46 % of the participants, around 20 % of the participants also had obtained university entrance qualification and around 33 % also had obtained a university degree or a comparable qualification.

A sensitivity analysis conducted using G*Power^51^ indicated that, with a sample size of *N* = 2586, four lineups per participant and α = β = 0.05, it was possible to detect effects of lineup position on the model parameters as small as *w* = 0.04 in goodness-of-fit tests with *df* = 5 (comparison of all lineup positions simultaneously, see Results section).

#### Ethics statement

Experiments 1 and 2 are part of a series of experiments that were approved by the ethics committee of the Faculty of Mathematics and Natural Sciences at Heinrich Heine University Düsseldorf. Both experiments were conducted in accordance with the Declaration of Helsinki. In both experiments, participants gave informed consent prior to participation. Participants were informed that the experiments involved seeing a video depicting verbal and physical harassment. They were asked not to proceed with the experiment if they felt uncomfortable imagining to watch such a video. At the end of the experiments, participants were informed that the video showing the crime had been staged.

#### Materials

We used the same staged-crime videos, suspect photos, filler photos and lineup design as in previous experiments^5,30,42–45^.

##### Staged-crime videos

Participants watched one of two staged-crime videos. In both videos, four culprits wearing fan clothing of the soccer club FC Bayern München harassed a victim wearing fan clothing of the soccer club Borussia Dortmund. All culprits were involved in the incident to a similar extent. By including four culprits, we were able to obtain four data points per participant and thereby increased the statistical sensitivity of our analyses. This procedure is ecologically valid as a notable share of real-world crimes includes multiple culprits^17,52,53^. Responding to multiple lineups after having witnessed a multiple-culprit crime seems to be harder than responding to a single lineup after having witnessed a single-culprit crime^54^.

The videos showed the culprits coming upon the victim at a bus stop and initially making fun of him and insulting him. Then they started to take his belongings and tossed them around. They began to attack the victim physically, ultimately knocking him to the ground. They continued to verbally and physically abuse him until one of the culprits noticed another person approaching (not visible in the videos). Then the culprits fled, shouting loudly. Both videos followed the same plot and included the same actions in the same sequence and timing. The videos only differed in the actors playing the culprits and the victim. The actors were selected ensuring that the actor for each culprit in Video A resembled the actor for the corresponding culprit in Video B. The videos showed a clear and frontal view of all the culprits’ faces. The culprits were visible for essentially the entire duration of the videos. Each video lasted for roughly 130 seconds and was shown at a resolution of 885 × 500 pixels.

##### Lineups

Participants were shown four lineups, one for each culprit in the staged-crime videos. Each lineup consisted of one suspect and five fillers. Culprit presence was manipulated using the crossed-lineup procedure^5,30,42–45^. In culprit-present lineups, the suspect was a culprit from the video that the participants had previously seen. In culprit-absent lineups, the suspect was a culprit from the video the participants had not seen. For example, if participants had seen Video A and the culprit-present lineups featured Culprits 1 and 3 from Video A, then the culprit-absent lineups featured innocent suspects that were Culprits 2 and 4 from Video B. The filler photos were obtained from the Center for Vital Longevity Face Database^55^. The photos showed white men between 18 and 29 years of age without any distinguishing marks such as scars or facial tattoos. The fillers were selected to resemble the culprits, who were white male young adults, in terms of body shape, hair color and hairstyle. All filler and suspect photos showed a frontal view of the face with a neutral facial expression. They were matched for lighting and face size and presented at a resolution of 142 × 214 pixels. The crossed-lineup procedure ensures that there are no systematic differences between culprits and innocent suspects but at the same time maintains ecological validity because, in police practice, the photo of the suspect, whose guilt or innocence is unknown, is often derived from a different source (e.g., a mug shot or social media) than the photos of the fillers (usually obtained from databases).

##### Procedure

The procedure followed the one described by Bell et al.^44^, Menne et al.^5,42^, Therre et al.^45^ and Winter et al.^30,43^. The experiment was programmed and conducted online using SoSci Survey^56^.

Participants were asked for their informed consent after having been informed that the experiment involved seeing a video depicting verbal and physical harassment. They were asked not to proceed if they felt uncomfortable imagining to watch such a video. Then, participants filled out the socio-demographic questionnaire and saw one of the two staged-crime videos described above. While watching the video, participants were unaware that they later would have to respond to lineups.

Which of the two videos was shown was determined at random. Participants started the video by clicking on a “Start” button. It was not possible to fast-forward, pause or replay the video. After the whole video had played, participants could continue to the next page which contained an attention-check question: Out of ten alternatives, participants had to correctly select “soccer fans” as the type of persons shown in the video. For participants who did not answer this question correctly, the experiment was terminated, and no further data were collected.

Next, the remaining participants received the lineup instructions. They were informed that they were about to see multiple lineups and were tasked with identifying the culprits from the video. Participants were told that the lineups might or might not contain a culprit and that it was as important to identify the culprit when he was present as it was to reject the lineup when the culprit was absent. Then, four lineups, one for each culprit in the video, were presented in a random order. Two of the lineups were randomly selected to be culprit-present lineups while the other two were culprit-absent lineups. As in studies by Clark and Davey^48 ^and by Meisters et al.^28^, all photos of the lineup members were presented simultaneously in one single row, which is one possible format to present simultaneous lineups^13^. This format approximates the layout of in-person lineups^22 ^which are still present in guidelines in different jurisdictions^13^. Furthermore, this presentation format ensures that lineup position varies in only one dimension (i.e., lineup position varies horizontally), just like in sequential lineups where individual photos are presented one after another (i.e., lineup position varies temporally).

The positions of the lineup members within each of the four lineups were completely randomized. Beneath each of the six photos was a button labeled “Yes, was present” that could be used to identify a lineup member. Additionally, a button “No, none of these persons was present” was displayed next to the lineup. This button could be used to reject the lineup. Participants had to either identify a lineup member or reject the lineup before they could click the “Next” button to proceed. After the last lineup, participants were debriefed and thanked for their participation.

### Results

Goodness-of-fit tests and parameter estimates were calculated using multiTree^57^. For data analysis, we needed six instances of the 2-HT eyewitness identification model depicted in Figure 1, one for each lineup position. The observed response frequencies that formed the basis of our analyses are reported in Table 1. The frequencies of culprit and innocent-suspect identifications refer to the number of culprits and innocent suspects that were identified in each lineup position in culprit-present and culprit-absent lineups, respectively. Similarly, the frequencies of filler identifications in culprit-present and culprit-absent lineups refer to the number of fillers that were identified in the respective lineup position. The frequencies of lineup rejections refer to the number of rejections of culprit-present and culprit-absent lineups in which the culprits or innocent suspects were placed in the respective lineup position. The guessing-based-selection parameter *g* of the 2-HT eyewitness identification model thus refers to the probability of selecting a lineup member based on guessing in each lineup position. Because the lineup positions of all six lineup members were completely randomized, guessing-based selection results in the selection of the suspect with the probability given by the reciprocal of the lineup size. Therefore, the constant 1$\:\div$* lineup size* was set to 0.16667, an approximation of the reciprocal of the lineup size.

**Table 1:** Observed response frequencies and proportions (in parentheses) as a function of lineup position in Experiment 1 (simultaneous lineups). The frequencies and proportions of culprit and innocent-suspect identifications refer to the number of culprits and innocent suspects that were identified in each lineup position in culprit-present and culprit-absent lineups, respectively. Similarly, the frequencies and proportions of filler identifications in culprit-present and culprit-absent lineups refer to the number of fillers that were identified in the respective lineup position. The frequencies and proportions of false and correct lineup rejections refer to the rejections of the culprit-present and culprit-absent lineups in which culprits or innocent suspects were placed in the respective lineup position. The proportions are rounded to two decimal places and therefore do not always add up exactly to 1.

	Lineup position					
	1	2	3	4	5	6
**Culprit-present lineups**						
Culprit identifications	313 (0.18)	290 (0.17)	275 (0.16)	242 (0.14)	285 (0.17)	289 (0.17)
Filler identifications	269 (0.21)	229 (0.18)	213 (0.16)	200 (0.15)	179 (0.14)	207 (0.16)
False lineup rejections	371 (0.17)	353 (0.16)	372 (0.17)	356 (0.16)	345 (0.16)	384 (0.18)
**Culprit-absent lineups**						
Innocent-suspect identifications	113 (0.19)	97 (0.17)	93 (0.16)	88 (0.15)	87 (0.15)	107 (0.18)
Filler identifications	359 (0.22)	251 (0.15)	263 (0.16)	265 (0.16)	244 (0.15)	273 (0.16)
Correct lineup rejections	462 (0.16)	511 (0.17)	468 (0.16)	476 (0.16)	535 (0.18)	480 (0.16)

**Figure 2:**
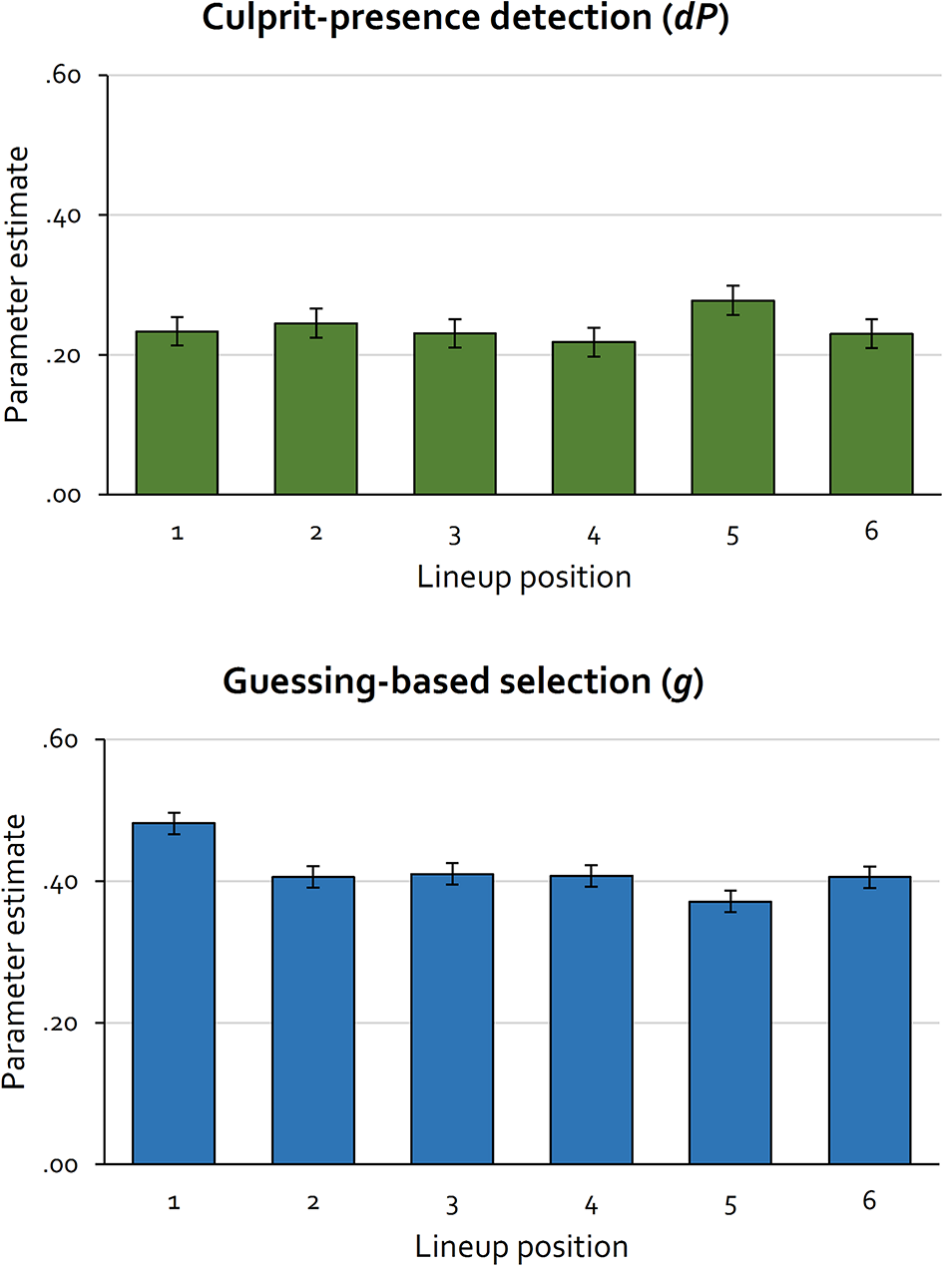
Estimates of parameters *dP* (culprit-presence detection; upper panel) and *g* (guessing-based selection; lower panel) as a function of lineup position in Experiment 1 (simultaneous lineups). The error bars represent standard errors.

The first step in applying a multinomial processing tree model is to find a base model that accurately describes the data. Model fit is evaluated by performing a goodness-of-fit test to assess the discrepancy between the observed data and the data predicted by the model. The goodness-of-fit statistic *G*^2^ is approximately chi-square distributed with degrees of freedom indicated in parentheses. If a goodness-of-fit test is non-significant, this attests that the model fits the data. In the base model, parameter *dA* was set to be equal across all lineup positions as the detection of the absence of the culprit was not expected to vary with lineup position. This base model fit the data, *G*^2^(5) = 6.83, *p* = .234. Parameter *dA* was estimated to be 0.02 (*SE* = 0.03). Parameter estimates for the culprit-presence-detection parameter *dP* and the guessing-based-selection parameter *g* are displayed in Fig. 2.

Multinomial processing tree models allow for testing hypotheses directly at the level of the parameters representing the cognitive processes underlying the observable behavior^32,46^. For example, the hypothesis that culprit-presence detection differs as a function of lineup position can be tested by restricting parameter *dP* to be equal across the six lineup positions. This equality restriction generates five degrees of freedom. If this equality restriction significantly decreases the model’s fit to the data relative to the fit of the base model, it can be concluded that culprit-presence detection differs as a function of lineup position. Restricting the culprit-presence-detection-parameter *dP* to be equal across all lineup positions did not significantly decrease the model fit, ∆*G*^2^(5) = 5.09, *p* = .405. This leads to the conclusion that the process of culprit-presence detection does not differ as a function of lineup position.

In contrast, restricting the guessing-based-selection parameter *g* to be equal across all lineup positions significantly decreased the model fit, ∆*G*^2^(5) = 35.98, *p *< .001. When each position was compared to each of the other lineup positions using Bonferroni-Holm-adjusted alpha levels to control for alpha error accumulation^58^, guessing-based selection differed significantly between Position 1 and each of the other lineup positions. Beyond that, there were no significant differences in guessing-based selection for all other pairwise comparisons of lineup positions (see Table 2). This leads to the conclusion that lineup members placed in Position 1 run a higher risk of being selected based on guessing than lineup members placed in other positions.

Parameter estimates for the biased-suspect-selection parameter b are shown in Table 3. Biased suspect selection did not differ significantly across lineup positions, ∆*G*^2^(5)= 1.95, *p* = .856. Descriptively, the estimates are low which reflects the fact that the lineups were constructed to be fair.

**Table 2:** Test statistics, *p*-values and the respective Bonferroni-Holm-adjusted alpha levels for the tests comparing guessing-based selection at each lineup position to guessing-based selection at each of the other lineup positions in Experiment 1. Comparisons are sorted by *p*-value and comparisons with statistically significant differences are typeset in bold. *b* Table 3. B ∆*G*^2^(5) = 1.95, *p*

Lineup-position comparison	∆*G*^2^(1)	*p*	Bonferroni-Holm-adjusted alpha level
**1 vs. 5**	**32.21**	**< 0.001**	**0.003 (0.05** $\:\div$ **15)**
**1 vs. 6**	**15.49**	**< 0.001**	**0.004 (0.05** $\:\div$ **14)**
**1 vs. 2**	**15.35**	**< 0.001**	**0.004 (0.05** $\:\div$ **13)**
**1 vs. 4**	**14.60**	**< 0.001**	**0.004 (0.05** $\:\div$ **12)**
**1 vs. 3**	**13.46**	**< 0.001**	**0.005 (0.05** $\:\div$ **11)**
3 vs. 5	3.90	0.048	0.005 (0.05 $\:\div$ 10)
4 vs. 5	3.26	0.071	0.006 (0.05 $\:\div$ 9)
2 vs. 5	3.10	0.078	0.006 (0.05 $\:\div$ 8)
5 vs. 6	3.04	0.081	0.007 (0.05 $\:\div$ 7)
3 vs. 6	0.06	0.809	0.008 (0.05 $\:\div$ 6)
2 vs. 3	0.05	0.821	0.01 (0.05 $\:\div$ 5)
3 vs. 4	0.03	0.870	0.013 (0.05 $\:\div$ 4)
4 vs. 6	0.01	0.939	0.017 (0.05 $\:\div$ 3)
2 vs. 4	0.00	0.951	0.025 (0.05 $\:\div$ 2)
2 vs. 6	0.00	0.988	0.05 (0.05 $\:\div$ 1)

**Table 3:** Estimates and standard errors (in parentheses) of parameter *b* (biased suspect selection) as a function of lineup position in simultaneous lineups in Experiment 1.

	Lineup position					
	**1**	**2**	**3**	**4**	**5**	**6**
Biased suspect selection	0.05 (0.01)	0.05 (0.01)	0.05 (0.01)	0.04 (0.01)	0.04 (0.01)	0.06 (0.01)

### Discussion

The model-based analyses of the data of Experiment 1 lead to the conclusion that lineup position has no effect on the detection-based processes underlying eyewitness responses in simultaneous lineups. Participants were just as likely to detect the presence of the culprit when he was placed in edge positions as when he was placed in middle positions. This finding is consistent with most previous research showing that lineup position has no effect on the rate of culprit identifications or the ability to discriminate between culprits and innocent suspects in simultaneous lineups^6,28,48^. These results are also in line with Palmer et al.’s^21^ proposition that participants are motivated to make accurate decisions and therefore attend the photos in all lineup positions equally closely. In the model-based analysis of guessing-based selection, there was no evidence that participants were more likely to select lineup members placed in middle positions based on guessing. Instead, guessing-based selection was increased for the lineup member placed in Position 1, the leftmost position of the lineup, with no pairwise differences between the other lineup positions. As a side note, this finding is already evident in the raw response frequencies (Table 1) as participants made more selections of suspects and fillers combined in Position 1 (1054 selections) than in any other position (≤ 876 selections in every other position). This result is consistent with findings showing that people have an increased tendency to select lineup members that are placed earlier in the reading direction compared to lineup members placed later^21,49,50^.

## Experiment 2

In Experiment 2, we investigated the effects of lineup position on the detection-based and non-detection-based processes in sequential lineups in which the lineup members are presented one after another. Sequential lineups were first proposed by Lindsay and Wells in 1985 as an alternative to simultaneous lineups^59 ^and have since been included in guidelines for lineups in various jurisdictions^13,60,61^.

As in Experiment 1, the first aim of Experiment 2 was to test whether lineup position affects the detection of the presence of the culprit. A priori, it was unclear whether, and, if so, how, lineup position might affect the detection of the presence of the culprit in sequential lineups. On the one hand, as more and more faces are shown, participants may gain more insight into the detection task, which could help their performance^62^. On the other hand, interference may build up due to the increasing number of faces seen previously in the lineup that might act as visually similar distractors^63^. Empirically, many studies have found no effect of lineup position on the rate of culprit identifications or the ability to discriminate between culprits and innocent suspects in sequential lineups^7,20,23,47,59,64^. In contrast, there are also some studies showing effects of lineup position in sequential lineups, but these reports are inconsistent. Some studies suggest that there is an advantage of placing the suspect in earlier positions^6,48,65 ^while others suggest that there is an advantage of placing the suspect in later positions^24,28,49,66^.

The second aim of Experiment 2 was to test whether lineup position affects guessing-based selection. In several previous studies, lineup position had no effect on overall identification rates, rejection rates or the response criterion in sequential lineups^23,59,64^. However, there are also studies suggesting that people are more likely to select lineup members in earlier positions^27,49,50 ^or later positions^28,65^. It was thus an open question as to whether the effects of Experiment 1—increased guessing-based selection in Position 1 with no differences between the other lineup positions— would be replicated in Experiment 2.

### Method

#### Participants

Participants were recruited and compensated through the research panel of Bilendi GmbH (https://www.bilendi.de). Of the data files of the participants who filled out the socio-demographic questionnaire at the beginning of Experiment 2, a total of 476 were excluded because the participants either did not complete the experiment or revoked the consent to the use of their data, 132 were excluded because the participants saw the staged-crime video more than once and 44 were excluded because the participants failed the attention check. The final sample included 2581 participants (1141 female, 1438 male, 2 diverse) with a mean age of 54 years (*SD *= 16). The sample was characterized by a diversified level of education: Secondary education had been completed by around 46 % of the participants, around 21 % of the participants also had obtained university entrance qualification and around 33 % also had obtained a university degree or a comparable qualification. A sensitivity analysis conducted using G*Power^51^ indicated that, with 6179 data points (fewer than to be expected based on the number of participants because only lineups with at most one identification were included in the data analysis, see Procedure section) and α = β = 0.05, it was possible to detect effects of lineup position on the model parameters as small as *w* = 0.06 in goodness-of-fit tests with *df* = 5 (comparison of all lineup positions simultaneously, see Results section).

### Materials

Materials were the same as those used in Experiment 1.

#### Procedure

The procedure was the same as the one used in Experiment 1 except that lineups were presented sequentially instead of simultaneously. In the sequential lineups, photos of the lineup members were presented one at a time in a randomized order. Participants had to decide for each lineup member whether he was a culprit or not. They could click the button “Yes, was present” underneath the photo or the button “No, this person was not present” to indicate their decision. Once participants had made their decision, they could proceed to the next lineup member by clicking the “Next” button. Irrespective of their responses, participants were shown all six lineup members. After the sixth lineup member, the lineup ended. If participants did not select any of the lineup members, the lineup was classified as rejected.

In sequential lineups, it is possible that eyewitnesses identify more than one lineup member, in which case the lineup administrators must decide on how to interpret the eyewitnesses’ identifications. In the past, researchers often used instructions emphasizing that only the first identification in the lineup would count^4,25,49,67 ^to evade this issue. In some studies, participants did not even see the remaining lineup members after having made an identification because the lineup was terminated immediately^6,7,47,64^. This is contrary to how lineups are conducted in the real world^68^. Lineup administrators likely want to avoid terminating the lineup procedure before the suspect’s face has been shown. Furthermore, defense lawyers could argue that the lineup consisted of fewer than the required number of lineup members if a lineup was terminated early. Therefore, jurisdictions^60,61 ^often require lineup administrators to always show all lineup members. In this case, one could argue that research should follow standard police procedure in order to arrive at valid conclusions^43,68^. With respect to position effects, Horry et al.^68^ have pointed out that the first-yes-counts rule alone could induce a position effect in the response rates by inflating the rate of initial identifications being accepted as valid. Counting the last identification under the reasoning that it revises earlier ones might cause position effects in the opposite direction. Since the aim of this experiment was to investigate position effects in sequential lineups as employed in police practice, we did not give first-yes-counts instructions. Additionally, because we aimed to investigate position effects on the cognitive processes underlying eyewitness responses instead of the consequences of decisions on how to analyze the data, we followed the approach of previous studies^69–71^ of only including lineups with no more than one identification (around 60 %) in our analyses.

## Results

**Figure 3:**
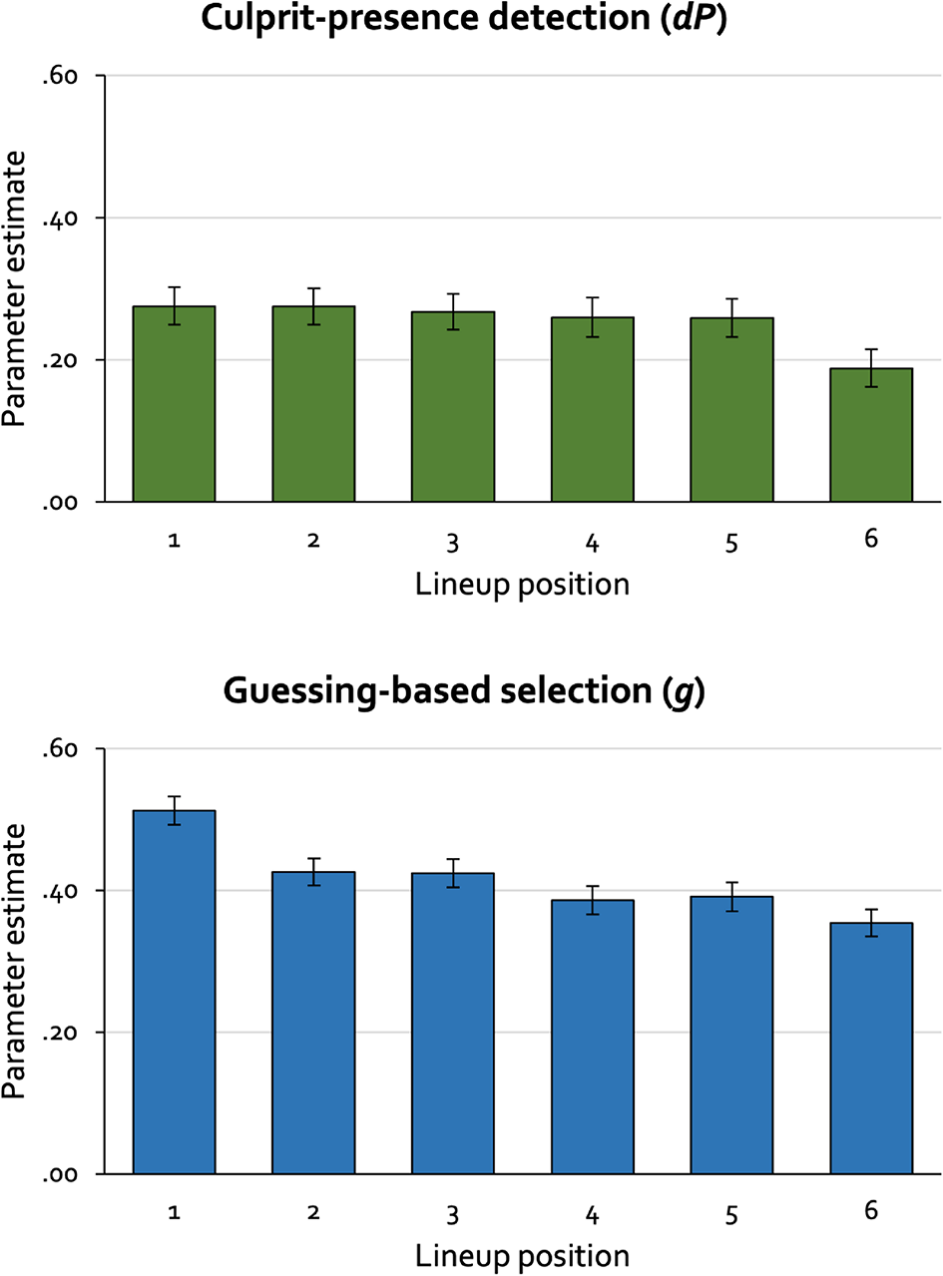
Estimates of parameters *dP* (culprit-presence detection; upper panel) and *g* (guessing-based selection; lower panel) as a function of lineup position in Experiment 2 (sequential lineups). The error bars represent standard errors.

The observed response frequencies that formed the basis of our analyses are reported in Table 4. We used the same base model as in Experiment 1. This base model fit the data, *G*^2^(5) = 6.79, *p* = .237. Parameter *dA* was estimated to be 0.00 (*SE* = 0.03). Parameter estimates for the culprit-presence-detection parameter *dP* and the guessing-based-selection parameter *g* are displayed in Figure 3.

Parallel to the results of Experiment 1, restricting the culprit-presence-detection parameter *dP* to be equal across all lineup positions did not significantly decrease the model fit, ∆*G*^2^(5) = 7.88, *p* = .163. This leads to the conclusion that the process of culprit-presence detection does not differ as a function of lineup position. Also parallel to the results of Experiment 1, restricting the guessing-based-selection parameter *g* to be equal across all lineup positions significantly decreased the model fit, ∆*G*^2^(5) = 49.07, *p *< .001. As in Experiment 1, each position was compared to each of the other positions using Bonferroni-Holm-adjusted alpha levels to control for alpha error accumulation^58^. Guessing-based selection differed significantly between Position 1 and each of the other positions. Beyond that, there were no significant differences in guessing-based selection for all other pairwise comparisons of lineup positions, except that guessing-based selection differed significantly between Position 2 and Position 6 (see Table 5). This leads to the conclusion that lineup members placed in Position 1 run a higher risk of being selected based on guessing than lineup members placed in other positions.

Parameter estimates for the biased-suspect-selection parameter b are shown in Table 6. Biased suspect selection did not differ significantly across lineup positions, ∆*G*^2^(5) = 10.90, p = .053. Descriptively, the estimates are low which reflects the fact that the lineups were constructed to be fair.

**Table 4:** Observed response frequencies and proportions (in parentheses) as a function of lineup position in Experiment 2 (sequential lineups). The frequencies and proportions of culprit and innocent-suspect identifications refer to the number of culprits and innocent suspects that were identified in each lineup position in culprit-present and culprit-absent lineups, respectively. Similarly, the frequencies and proportions of filler identifications in culprit-present and culprit-absent lineups refer to the number of fillers that were identified in the respective lineup position. The frequencies and proportions of false and correct lineup rejections refer to the rejections of the culprit-present and culprit-absent lineups in which culprits or innocent suspects were placed in the respective lineup position. The proportions are rounded to two decimal places and therefore do not always add up exactly to 1.

	Lineup position					
	1	2	3	4	5	6
**Culprit-present lineups**						
Culprit identifications	227 (0.23)	194 (0.20)	151 (0.15)	161 (0.16)	136 (0.14)	119 (0.12)
Filler identifications	185 (0.25)	138 (0.19)	122 (0.17)	104 (0.14)	95 (0.13)	89 (0.12)
False lineup rejections	190 (0.15)	215 (0.18)	194 (0.16)	199 (0.16)	194 (0.16)	234 (0.19)
**Culprit-absent lineups**						
Innocent-suspect identifications	82 (0.24)	65 (0.19)	38 (0.11)	67 (0.20)	40 (0.12)	49 (0.14)
Filler identifications	225 (0.21)	198 (0.18)	174 (0.16)	173 (0.16)	161 (0.15)	151 (0.14)
Correct lineup rejections	280 (0.15)	330 (0.18)	290 (0.16)	331 (0.18)	286 (0.16)	292 (0.16)

**Table 5:** Test statistics, *p*-values and the respective Bonferroni-Holm-adjusted alpha levels for the tests comparing guessing-based selection at each lineup position to guessing-based selection at each of the other lineup positions in Experiment 2. Comparisons are sorted by *p*-value and comparisons with statistically significant differences are typeset in bold.*b* are shown in table 6.

Lineup-position comparison	∆*G*^2^(1)	*p*	Bonferroni-Holm-adjusted alpha level
**1 vs. 4**	**26.34**	**< 0.001**	**0.003 (0.05** $\:\div$ **15)**
**1 vs. 5**	**23.20**	**< 0.001**	**0.004 (0.05** $\:\div$** 14)**
**1 vs. 6**	**40.24**	**< 0.001**	**0.004 (0.05** $\:\div$ **13)**
**1 vs. 2**	**12.90**	**< 0.001**	**0.004 (0.05** $\:\div$ **12)**
**1 vs. 3**	**12.73**	**< 0.001**	**0.005 (0.05** $\:\div$ **11)**
**2 vs. 6**	**8.39**	**0.004**	**0.005 (0.05** $\:\div$ **10)**
3 vs. 6	7.49	0.006	0.006 (0.05 $\:\div$ 9)
2 vs. 4	2.65	0.104	0.006 (0.05 $\:\div$ 8)
3 vs. 4	2.25	0.134	0.007 (0.05 $\:\div$ 7)
5 vs. 6	2.03	0.155	0.008 (0.05 $\:\div$ 6)
2 vs. 5	1.95	0.162	0.01 (0.05 $\:\div$ 5)
3 vs. 5	1.64	0.200	0.013 (0.05 $\:\div$ 4)
4 vs. 6	1.61	0.205	0.017 (0.05 $\:\div$ 3)
4 vs. 5	0.03	0.853	0.025 (0.05 $\:\div$ 2)
2 vs. 3	0.01	0.935	0.05 (0.05 $\:\div$ 1)

**Table 6:** Estimates and standard errors (in parentheses) of parameter *b* (biased suspect selection) as a function of lineup position in sequential lineups in Experiment 2.

	Lineup position					
	**1**	**2**	**3**	**4**	**5**	**6**
Biased suspect selection	0.06 (0.02)	0.04 (0.01)	0.01 (0.01)	0.06 (0.02)	0.02 (0.01)	0.04 (0.01)

## Discussion

Parallel to the results of Experiment 1, participants showed the same ability to detect the presence of the culprit, no matter the position he was placed in. This conclusion is in line with several previous studies showing that lineup position had no effect on the rate of culprit identifications or the ability to discriminate between culprits and innocent suspects in sequential lineups^7,20,23,47,59,64^. These findings suggest that participants attended to the photos of all lineup members equally closely regardless of their position in the lineup.

In the model-based analysis of guessing-based selection, significant effects of lineup position were found. Specifically, guessing-based selection was increased for the lineup member placed in Position 1, with no consistent differences observed between the other lineup positions. As a side note, this finding is already evident in the raw response frequencies (Table 4) as participants made more selections of suspects and fillers combined in Position 1 (719 selections) than in any other position (≤ 595 selections in every other position). Such a pattern, in turn, is in line with those previous studies that have found that people are more likely to select lineup members in earlier positions^27,49,50^. The observed effect of lineup position on guessing-based selection in sequential lineups is thus parallel to the effect of lineup position on guessing-based selection in simultaneous lineups observed in Experiment 1.

## General discussion

When compiling lineups, practitioners as well as researchers often avoid, or recommend to avoid, placing the suspect in Position 1 of a lineup^2,15–20^. However, so far it has been unclear whether this hesitance to place the suspect in the first position of a lineup is justified. Here the 2-HT eyewitness identification model^30,31 ^was used to investigate how lineup position affects the detection-based and non-detection-based processes underlying eyewitness responses. To allow for valid conclusions, the lineup members were randomly assigned to each of the six lineup positions. Furthermore, large sample sizes were used to attain many data points for each of the lineup positions, ensuring a high sensitivity of the statistical tests assessing the effects of lineup position. This made it possible to examine the cognitive processes underlying eyewitness responses in each lineup position separately, without limiting the analyses to certain lineup positions or aggregating over lineup positions. To achieve a comprehensive assessment of the effects of lineup position, Experiment 1 served to investigate effects of lineup position in simultaneous lineups and Experiment 2 was focused on sequential lineups. Our model-based analyses of lineup-position effects revealed a consistent pattern for both types of lineups. First, there were no lineup-position effects on the ability to detect the presence of the culprit, neither in simultaneous nor in sequential lineups. These results are in line with previous research showing no effects of lineup position on the rate of culprit identifications or the ability to discriminate between culprits and innocent suspects in simultaneous lineups^6,28,48 ^and sequential lineups^7,20,23,47,59,64^. These findings lead to the conclusion that participants attended to all photos equally closely regardless of the lineup position in which the photos were presented.

However, in both simultaneous and sequential lineups, participants were significantly more likely to make a guessing-based selection of the lineup member placed in Position 1—a particularly salient position in both simultaneous and sequential lineups—than of the lineup members placed in other positions. The finding that guessing-based selection is increased in Position 1 combined with the finding that there were no consistent differences between the other lineup positions at first glance validates the reluctance of lineup administrators to place the suspect in the first position^15^. If the suspect is innocent, the risk of a false identification due to guessing-based selection is increased. If the suspect is guilty, placing the suspect in first position may increase the risk that the lineup evidence is discarded in court due to the potentialy inflated influence of guessing-based selection in the identification of the suspect. A straightforward solution seems to be to avoid placing the suspect in Position 1 of lineups^2,15–20 ^to decrease the probability that the suspect is selected based on guessing. However, if eyewitnesses became aware that the suspect never appears in the first position, they could strategically disregard the lineup member presented in Position 1. This could reduce the functional size of the lineup and effectively turn Position 2 into the new Position 1, in which case the problem would not be solved at all. To remedy the problem, one should first try to achieve an even distribution of guessing-based selections of lineup members across all lineup positions, for example by informing witnesses that lineup members were assigned to positions randomly or by using an array shape without any particularly salient positions^21^. Second, one should try to reduce the probability of guessing-based selections as much as possible, for example by using lineup instructions that discourage guessing-based selection^43,45^.

Following studies by Clark and Davey^48 ^and Meisters et al.^28^, we displayed all lineup members in a single row in simultaneous lineups. This lineup format represents one possible format to present simultaneous photo lineups^13 ^that approximates the layout in in-person lineups. Furthermore, this lineup format simplifies the interpretation of the results by ensuring that lineup position changes in only one dimension in both simultaneous and sequential lineups where photos are presented in a horizontal and a temporal order, respectively. In contrast, presenting the simultaneous lineups in a two-dimensional array would introduce two spatial dimensions, the horizontal and the vertical dimension, complicating the interpretation of the results and differing more from sequential lineups. However, in some jurisdictions, for instance in the USA^19^, simultaneous photo lineups are presented as two-dimensional arrays such as two rows of three photos. It is unclear whether and how the effects of position in simultaneous lineups reported here generalize to this two-dimensional format, as it was not possible to examine position effects that may occur only in two-dimensional arrays, such as top-row biases, in the present Experiment 1. Therefore, further research is needed to examine the effects of position on culprit-presence detection and guessing-based selection in two-dimensional arrays (for analyses pertaining to identification rates and discriminability, see^21,29^).

Another limitation of the present research is that the analysis of sequential lineups included data only from those sequential lineups in which at most one identification was made. We thereby ensured that we analyzed the effects of lineup position on the cognitive processes rather than consequences of decisions on how to analyze the data. Anecdotally, we have learned from communications with chief inspectors responsible for conducting lineups at the Düsseldorf Police Department that it also occurs in practice that lineups with more than one identification are discarded as evidence, as the validity of such lineups would presumably be questioned by defense lawyers because at least one of the identifications is necessarily false. Nevertheless, this approach limits the generalizability of our findings to sequential lineups where a single identification is made. Specifically, our analyses do not address the effects of lineup position on the cognitive processes underlying eyewitness responses to lineups when more than one identification is made within the same sequential lineup.

## Conclusion

The aim of the present study was to gain a better understanding of how lineup position affects the latent cognitive processes underlying eyewitness responses. The widespread concerns about placing the suspect in the first position in lineups documented in the literature^2,15–20^ are validated by the present research. The present model-based analyses lead to the conclusion that lineup position had no effect on the detection of the presence of the culprit in simultaneous and sequential lineups. However, guessing-based selection of the lineup members differed as a function of lineup position in both simultaneous and sequential lineups. Specifically, lineup members placed in Position 1 faced a higher risk of being selected based on guessing than lineup members placed in other positions.

## Data Availability

The data of both experiments are available at the project page of the Open Science Framework under https://osf.io/w582g/.
